# Heterogeneous risk attitudes and waves of infection

**DOI:** 10.1371/journal.pone.0299813

**Published:** 2024-04-09

**Authors:** Daisuke Fujii, Taisuke Nakata, Takeshi Ojima

**Affiliations:** 1 Research Institute of Economy, Trade and Industry (RIETI), Chiyoda, Tokyo, Japan; 2 Graduate School of Economics, University of Tokyo, Bunkyo, Tokyo, Japan; 3 Faculty of Economics, Soka University, Hachioji, Tokyo, Japan; Texas A&M University College Station, UNITED STATES

## Abstract

Many countries have experienced multiple waves of infection during the COVID-19 pandemic. We propose a novel but parsimonious extension of the SIR model, a CSIR model, that can endogenously generate waves. In the model, cautious individuals take appropriate prevention measures against the virus and are not exposed to infection risk. Incautious individuals do not take any measures and are susceptible to the risk of infection. Depending on the size of incautious and susceptible population, some cautious people lower their guard and become incautious—thus susceptible to the virus. When the virus spreads sufficiently, the population reaches “temporary” herd immunity and infection subsides thereafter. Yet, the inflow from the cautious to the susceptible eventually expands the susceptible population and leads to the next wave. We also show that the CSIR model is isomorphic to the SIR model with time-varying parameters.

## 1 Introduction

**Fig 1:**
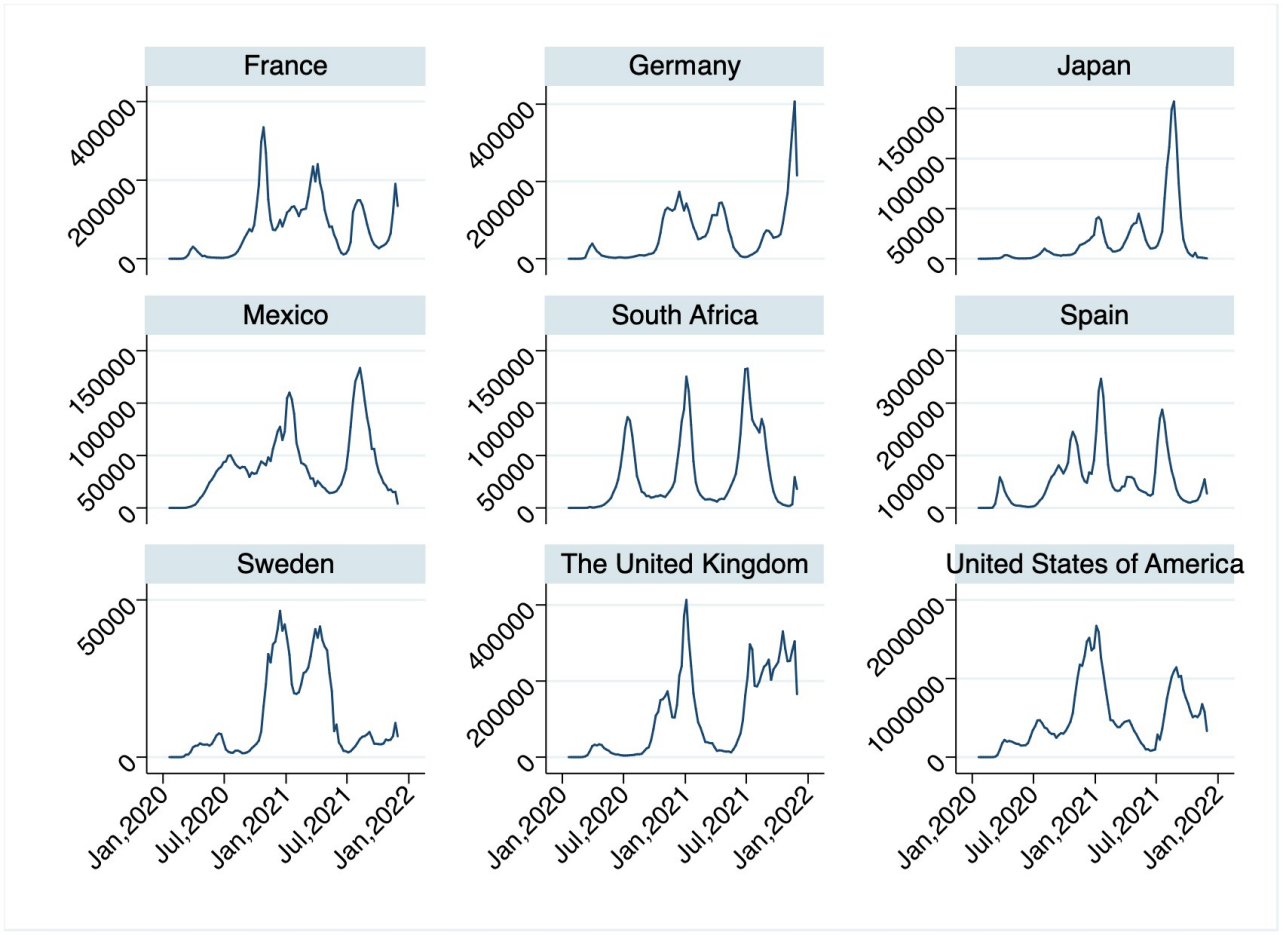
Weekly number of new COVID-19 cases between January 2020 and November 2021. (Source: WHO Coronavirus Dashboard).

The COVID-19 pandemic caused by a coronavirus SARS-CoV-2 rapidly spread around the globe posing unprecedented health and economic challenges to mankind. Since its outbreak, the pandemic has given rise to waves of infection in many countries, as shown in in Fig 1. The susceptible-infectious-removed (SIR) model, one of the most fundamental and widely-used epidemic models, cannot generate waves of infection in its purest form.

In this paper, we propose a novel modification to the SIR model, a “CSIR” model, to describe infection waves with constant population. The structure of our model is straightforward, yet it generates complex dynamics of infection. Key features of our CSIR model are that people have different risk attitudes toward the virus and that these attitudes vary over time. In the standard SIR model, those who have not been infected are homogeneous in terms of their risk attitudes.

Our model starts when a new virus emerges, all members of society learn about it, and some individuals become “cautious”. Those cautious individuals (compartment C) take appropriate measures against the virus—such as staying at home and wearing masks—and are not subject to infection risk. Susceptible individuals are less vigilant about the virus and vulnerable to infection. Depending on the size of the susceptible population, some cautious people lower their guard and become less vigilant against, and hence susceptible to, the virus. When the virus spreads, it eventually reaches “local” herd immunity and subsides thereafter. Nevertheless, the inflow from the cautious to the susceptible regenerates the expansion of the susceptible population leading to the next wave of infection. This repeated process creates a finite number of infection waves.

The multiplicity of waves in the CSIR model before reaching the steady state means that the size of each wave is smaller than the size of the wave in the SIR model when the parameter of the CSIR model is calibrated to match the final epidemic size in the SIR model. During the COVID-19 pandemic, the peak of many infection waves was substantially lower than what the standard SIR would predict, even after taking into account factors such as vaccination and NPIs. Our model provides a theory of why that was the case that relies on heterogeneity in risk attitudes and their time-variation.

In addition to its ability to generate waves, the CSIR model possesses other intriguing features that are distinct from those of the standard SIR model. For example, we show via numerical analysis that a higher transmission rate leads to lower peaks of early infection waves and higher peaks for late infection waves in the CSIR model. In the SIR model, a higher transmission rate leads a higher peak. We also show, analytically, that a higher transmission rate implies a lower epidemic final size in the CSIR model, whereas it implies a higher final size in the SIR model.

Existing literature has provided a variety of extensions to—and modifications of—the standard SIR model that can generate waves—or oscillations in the terminology often used in the literature. Some models focus on behavioral responses such as self-quarantine like ours. Other models focus on reinfection, age-structure, fear effects, seasonality, or network structures. At the same time, beyond the academic literature, there are several factors that might have contributed to the generation of waves during the COVID-19 pandemic, such as the periodic emergence of a more infectious variant of coronavirus SARS-CoV-2 and government lockdown policies. Our CSIR model featuring heterogeneous risk attitudes and their time-variation provides the literature with a novel mechanism for multiple waves as well as a plausible factor that might have contributed to the emergence of multiple waves during the COVID-19 pandemic. We review related literature and make our contribution clear in Section 5. We put the literature review in an unusual place since it is difficult to see the difference from other theories without presenting our model.

This paper is organized as follows. The next section reviews the standard SIR model and shows why waves do not occur in its purest form. Section 3 presents the CSIR model and conditions to generate infection waves. Analytic properties are also discussed. Section 4 highlights the difference between SIR and CSIR models when a more infectious virus emerges. Section 5 reviews related literature and Section 6 concludes.

## 2 Review of the SIR model

This section reviews the standard SIR model introduced in [1] and explains why it does not explain the waves of infection. Consider the following SIR model

$$\begin{array}{cc} \frac{dS(t)}{dt} & =-\beta S(t)I(t)\\ \frac{dI(t)}{dt} & =\beta S(t)I(t)-\gamma I(t)\\ \frac{dR(t)}{dt} & =\gamma I(t), \end{array}$$

where *S*, *I*, and *R* are susceptible, infectious, and removed populations respectively. Let *N*(*t*) = *βS*(*t*)*I*(*t*) denote the number of new cases at time *t*. The removed population can also be divided into recovered and dead classes, but we do not focus on the difference in this paper since *R*(*t*) does not affect the dynamics of the system. The parameter *β* is called a transmission rate, and governs the infectious force. *γ* denotes the removal rate, where 1/*γ* is the average duration of infectiousness. We normalize total population to one

S(t)+I(t)+R(t)=1

so that *S*, *I*, and *R* are corresponding population shares. Total population is preserved at any time since $\frac{dS}{dt}+\frac{dI}{dt}+\frac{dR}{dt}=0$. The basic reproduction number is defined as ${ℜ}_{0}=\frac{\beta }{\gamma }$. From the expression of $\frac{dI}{dt}$, if ℜ_0_ < 1, then *I*(*t*) is decreasing regardless of *S*(*t*) and there is no outbreak of infection. If ℜ_0_ > 1, an outbreak occurs. In that case, it is useful to focus on the effective reproduction number ℜ_*t*_ = ℜ_0_ × *S*(*t*). If ℜ_*t*_ > 1, infection keeps spreading whereas if ℜ_*t*_ < 1, the infectious population will decrease. Since *S*(*t*) is monotonically decreasing, when it crosses $\frac{1}{{ℜ}_{0}}=\frac{\gamma }{\beta }$, the pandemic will converge to an end. Hence, $\frac{1}{{ℜ}_{0}}$ is called the herd immunity threshold.

**Fig 2:**
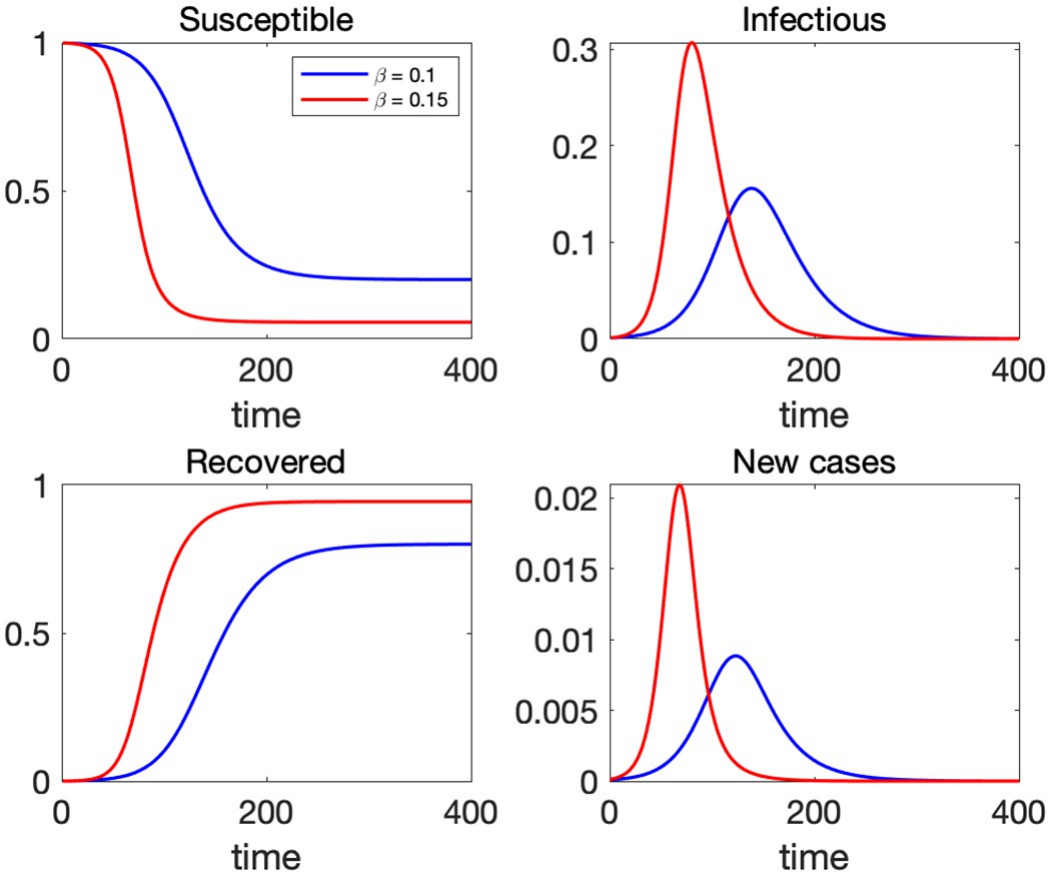
Time paths of each population in the SIR model with *γ* = 0.05 and *β* = 0.1 (blue) and *β* = 0.15 (red).

We discretize the model and interpret time at a daily frequency. Throughout this paper, we assume *γ* = 0.05 implying that the average infectious period is 20 days. Fig 2 depicts the time paths of *S*, *I*, *R* and *N* (new cases) for the initial values [*S*(0), *I*(0), *R*(0)] = [0.999, 0.001, 0] and two values of transmission rate: *β* = 0.1 and *β* = 0.15. In the graphs of *I* and *N*, we observe only one peak of infection in both cases. If the virus is more contagious (red line), the peak of infection is higher and comes earlier. Also, the cumulative number of infected people (*R*(∞)) will be higher if *β* is higher.

**Fig 3:**
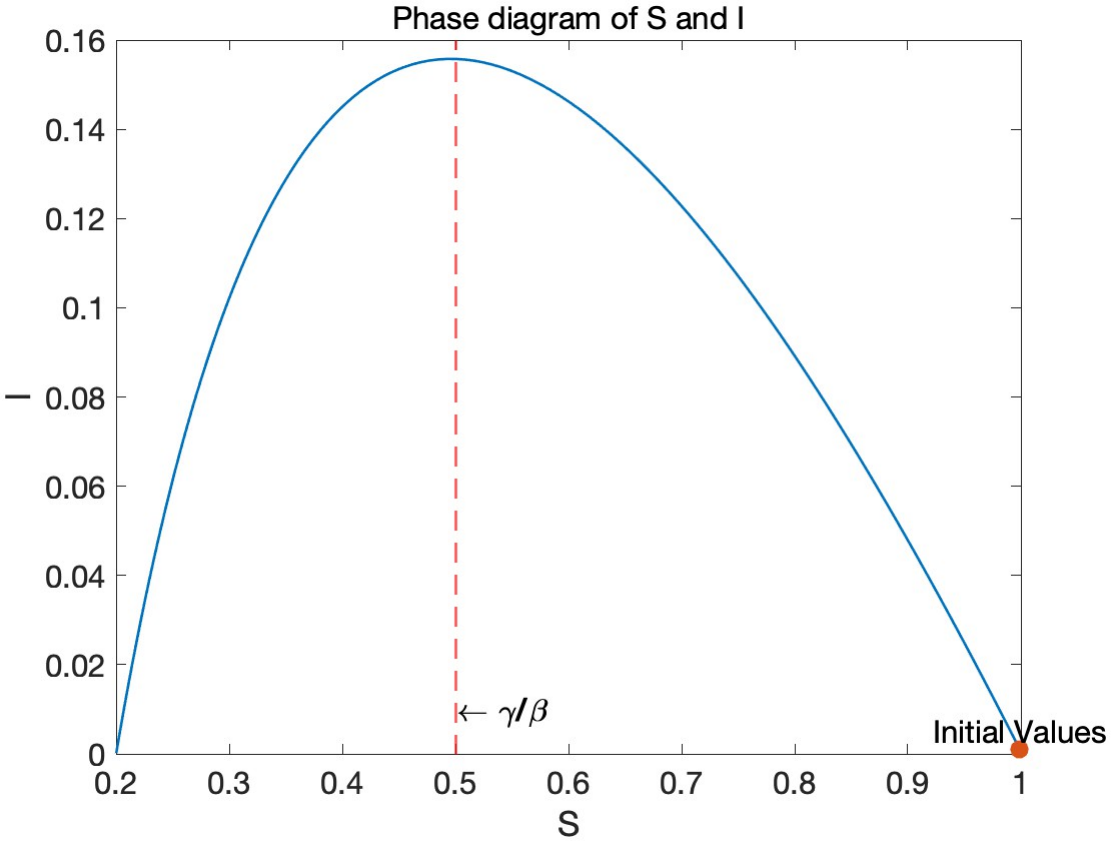
Phase diagram of the SIR model with *β* = 0.1 and *γ* = 0.05.

Fig 3 shows the phase diagram of *S* and *I* in the SIR model. The dynamics start from the bottom right corner. Since $\frac{\beta }{\gamma }>1$ and the number of susceptible people is large enough, infection spreads. *I*(*t*) goes up and *S*(*t*) goes down. When *S*(*t*) crosses $\frac{\gamma }{\beta }$, *I*(*t*) starts decreasing and the system will converge to the steady state with *I*(∞) = 0. Since *S*(*t*) is monotonically decreasing, after crossing the herd immunity threshold, it cannot generate the second wage of infection.

If we linearize the dynamic system around the steady state *I*(*t*) = 0, we obtain

$$[\begin{array}{c} S(t+1)\\ I(t+1) \end{array}]=[\begin{array}{cc} 1 & -\beta S\\ 0 & 1+\beta S-\gamma \end{array}][\begin{array}{c} S(t)\\ I(t) \end{array}].$$

The eigenvalues of the Jacobian matrix are λ_1_ = 1 and λ_2_ = 1 + *βS* − *γ*. Since they are not complex conjugate, we know that this system generates only one peak of infection.

As reviewed in Section 5, the existing studies have shown that waves can emerge in modified versions of the standard SIR model. In the sections that follow, we will present a novel modification that features heterogeneity in risk attitudes and their time-variation.

## 3 CSIR model and waves of infection

### 3.1 Model description

In this section, we present the CSIR model and its properties. As described in Section 1, we introduce a new class of population, the cautious people, to the standard SIR model. We will denote the groups of cautious people by “*C*.” The dynamics are described by the following system of differential equations:

$$\begin{array}{c} \frac{dC(t)}{dt}=-\alpha S(t)C(t) \end{array}$$ (1)

$$\begin{array}{c} \frac{dS(t)}{dt}=\alpha S(t)C(t)-\beta S(t)I(t) \end{array}$$ (2)

$$\begin{array}{c} \frac{dI(t)}{dt}=\beta S(t)I(t)-\gamma I(t) \end{array}$$ (3)

$$\begin{array}{c} \frac{dR(t)}{dt}=\gamma I(t). \end{array}$$ (4)

**Fig 4:**
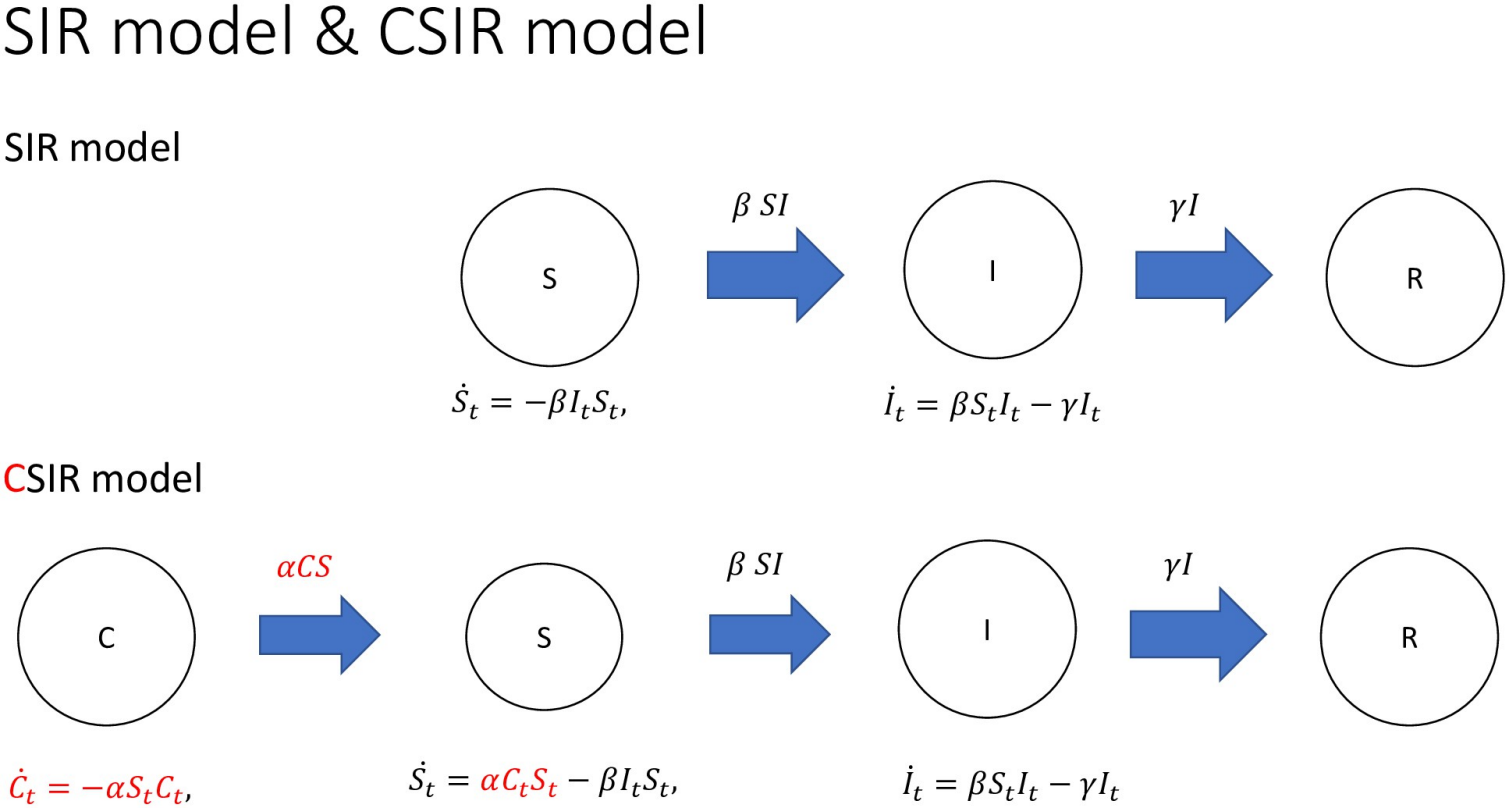
Flow chart of SIR and CSIR models.

In the model, cautious individuals (compartment *C*) take appropriate prevention measures against virus such as staying at home and are not exposed to infection risk. Susceptible individuals are incautious about virus and might get infected. In every period, some fraction of the cautious people lower their guard and become susceptible. The transition from *C* to *S* is modeled by *αS*(*t*)*C*(*t*). There is a peer effect of *S* on the outflow of *C*. The more incautious people go out into the town, the more cautious people relax their efforts and become incautious. We call *α* “a rate of slackening”. Unlike the SIR model, there are both inflow and outflow of *S*(*t*), which allow the volume of the susceptible to expand and shrink. The CSIR model resembles a prey-predator model, but the monotonically decreasing *C* generates a different model behavior over time. Flow charts of the SIR and CSIR models are summarized in Fig 4.

### 3.2 Waves of infection

**Fig 5:**
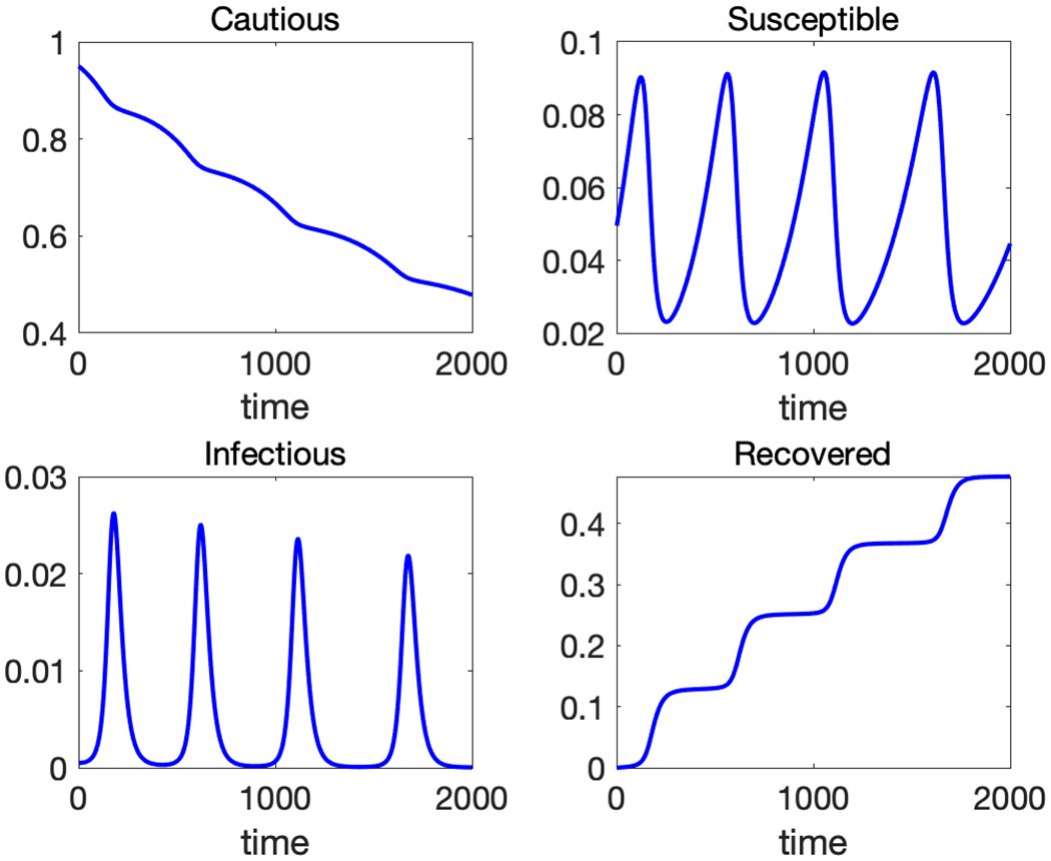
Time paths of *C*, *S*, *I* and *R* with *α* = 0.007, *β* = 1, and *γ* = 0.05.

Fig 5 depicts the time paths of the four variables with initial values *C*(0) = 0.95, *S*(0) = 0.0495, *I*(0) = 0.0005, *R*(0) = 0, and parameters [*α*, *β*, *γ*] = [0.007, 1, 0.05] for *T* = 2000. We assume that the news of an outbreak is shared by all individuals immediately so that 95% of people are cautious about the virus in the initial period. Around five percent of population does not fear the risk of infection. Gradually, some of the cautious people loosen infection control measures and flow into the susceptible class. Infection spreads among the susceptible and reaches its first peak around the 200th day (bottom-left panel of Fig 5). At this point, there are not enough susceptible people to spread infection further, and the volume of infected population starts to decline leading to “local” or “temporary” herd immunity. When the number of the infected remains at a low level for a while, the susceptible population expands because the inflow from the cautious class outweighs the outflow to the infected class. This expansion of the susceptible population results in the next wave of infection. The above process repeats over time and generates multiple waves of infection. In S1 Appendix, we consider alternative specifications of the flow between *C* and *S*. In particular, we allow a reverse flow from *S* to *C*, which might increase the population of *C* temporarily. Even in those cases, the emergence of waves is a general feature of the CSIR model as long as the population of cautious people exhibits an overall downward trend.

In the early phase of the COVID-19 pandemic, infection begun to drop at a much lower level than the herd immunity threshold predicted by the SIR model in many countries. It is widely accepted that non-pharmaceutical interventions (NPIs) such as stay-at-home orders and city-wide lockdowns played a key role to level off infection. We provide an alternative mechanism to explain the earlier and lower peak of infection. In the CSIR model, the spread of infection starts from a certain fraction of population who are incautious about virus, and once enough of them are infected, infection starts to decline. This threshold is much lower than that of the SIR model which assumes all agents are homogeneous and equally mixed. Yet, because risk attitudes of people change over time, the CSIR model generates multiple waves. It is necessary that *C*(0) is sufficiently large and *C*(*t*) gradually declines over time for the CSIR model to generate infection waves. This trend is related to the misperception of infection risks considered in the SIR-macro model of [2]. In their model, people are overly cautious in the early periods of pandemic but gradually let their guard down as more information becomes available to explain the actual path of infection in Japan.

**Fig 6:**
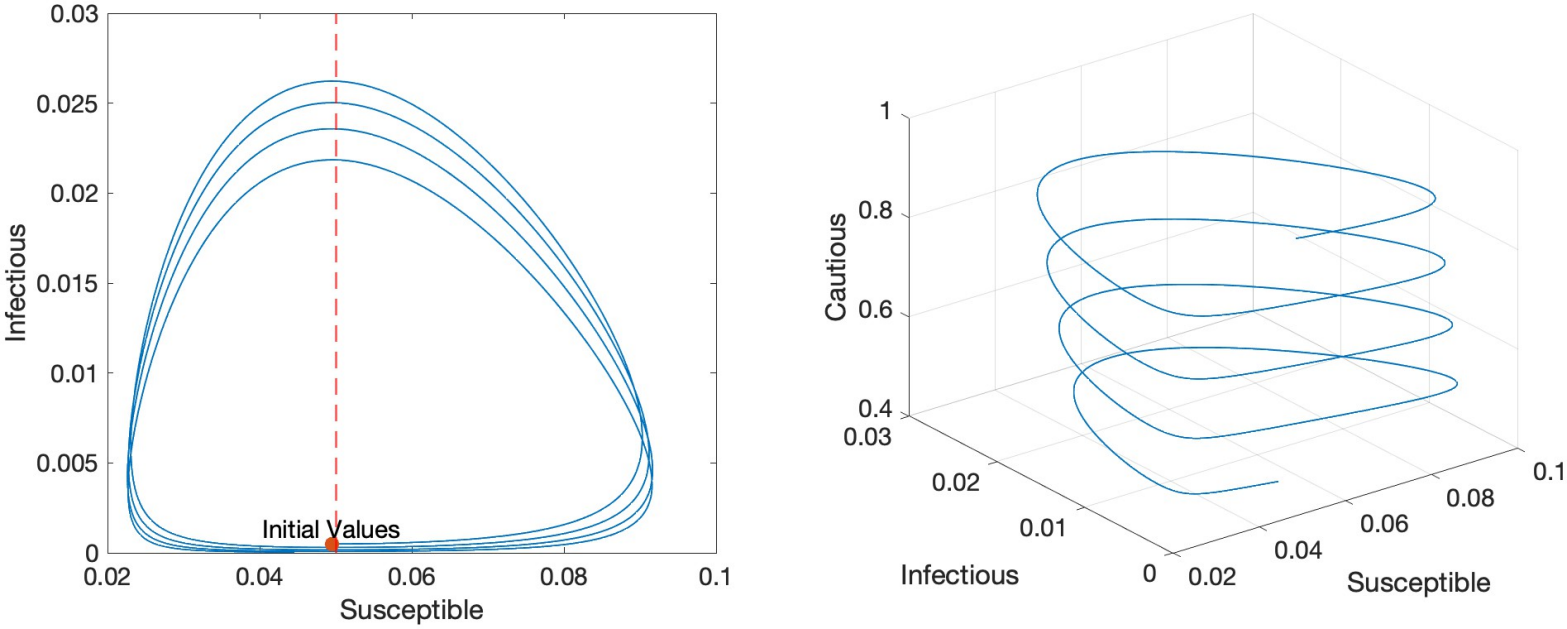
Phase diagrams of a CSIR model.

Fig 6 illustrates the phase diagrams of the CSIR model. The left panel is the phase diagram on a S-I plane. The dynamics start at the red dot and move counterclockwise. From the Eq (2), we know that *S* is increasing when $I<\frac{\alpha }{\beta }C$, and vice versa. Likewise from Eq (3), *I* is increasing when $S>\frac{\gamma }{\beta }$. At the initial point, *I* is so small that *S* starts increasing. Once *S* becomes larger than $\frac{\gamma }{\beta }$, *I* starts increasing (southeast part of the graph). When *I* becomes sufficiently large ($I>\frac{\alpha }{\beta }C$), *S* starts decreasing (northeast part of the graph). Once *S* crosses the temporary herd immunity threshold $\frac{\gamma }{\beta }$ depicted by a vertical red dotted line, *I* starts decreasing (northwest part of the graph). When *I* shrinks sufficiently, *S* starts increasing again (southwest part of the graph). This cycle repeats and gives rise to the waves of infection. The right panel of Fig 3 is a 3-D phase diagram in a S-I-C space. The dynamics start at the top and move down a helix. The left panel corresponds to an upper view of this 3-D phase diagram.

**Fig 7:**
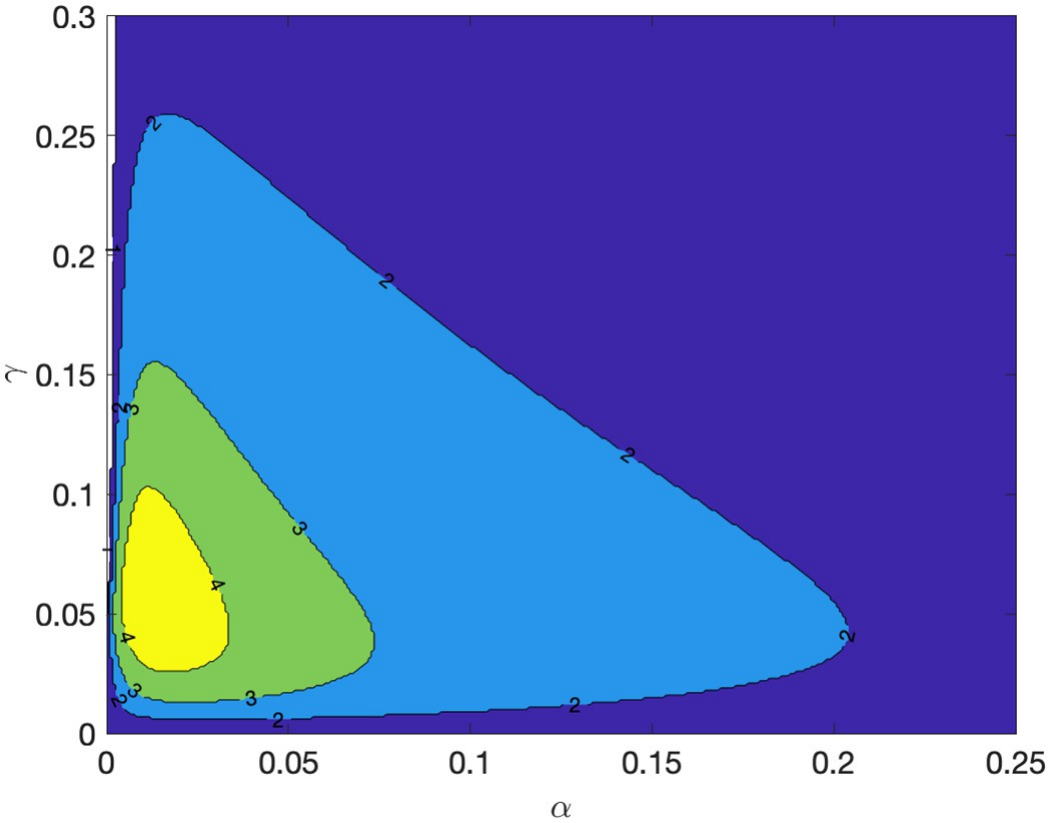
Contour plot for the number of waves for *T* = 2000 with different values of *α* and *γ*. Initial values are *C*(0) = 0.95, *S*(0) = 0.0495, *I*(0) = 0.0005, *R*(0) = 0 and *β* = 1.

Given an initial condition, the number of waves, defined as the number of peaks of *I*, generated by the CSIR system depends on parameter values. Fig 7 displays a contour plot for the number of waves within 2000 periods with different values of *α* and *γ* holding *β* = 1 and the same initial condition as in Fig 5. If *α* and *γ* are relatively small, four waves occur. When *α* is large, only one peak is observed because the inflow from *C* to *S* is too rapid that there will not be enough *C* left to generate the next wave after the first peak. As *α* becomes large, the CSIR model approaches to the SIR model since most of the population starts from the state *S*. A larger value of *γ* also implies a lower number of waves because the volume of *I* shrinks too quickly, and it will take time to pile up enough volume of *S* to generate the next wave.

**Fig 8:**
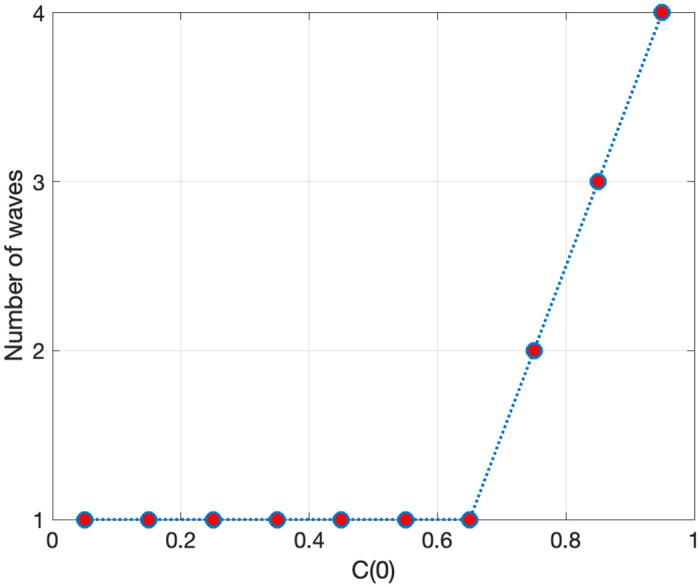
Initial population of the cautious *C*(0) and the number of waves.

The above analysis also reveals that the initial population of the cautious group must be large enough to generate multiple waves. Fig 8 plots the number of infection waves in the CSIR model for different initial values of *C*(0) ranging from 0.05 to 0.95. The parameters are [*α*, *β*, *γ*] = [0.05, 1, 0.07] and *T* = 2000. For each *C*(0), other initial values are set as *S*(0) = [1 − *C*(0)] × 0.99, *I*(0) = [1 − *C*(0)] × 0.01, and *R*(0) = 0. If *C*(0) is below 0.7, the model cannot generate any waves. The extreme case of *C*(0) = 0 collapses to the SIR model, so there would not be any waves as shown in the previous section.

### 3.3 Steady-state paths

Unlike the SIR model, the CSIR model described by Eqs (1)–(4) does not reach to the steady-state level of *S*. Nonetheless, further analytical investigations are possible by considering a new variable, and they provide more insights into the CSIR model.

Let $K_{t}=\frac{I_{t}}{C_{t}}$. The law of motion of this new variable is given by

$$\frac{dK_{t}}{dt}=(\alpha +\beta )K_{t}S_{t}-\gamma K_{t}.$$

We have

$$\begin{array}{cc} dK_{t} & =d(\frac{I_{t}}{C_{t}})=\frac{dI_{t}C_{t}-I_{t}dC_{t}}{C_{t}^{2}}=\frac{\left(\beta S_{t}I_{t}-\gamma I_{t})C_{t}-I_{t}(-\alpha S_{t}C_{t}\right)}{C_{t}^{2}}dt\\ & =\frac{(\beta S_{t}I_{t}-\gamma I_{t})+\alpha I_{t}S_{t}}{C_{t}}dt=[\beta (\frac{I_{t}}{C_{t}})S_{t}-\gamma (\frac{I_{t}}{C_{t}})+\alpha (\frac{I_{t}}{C_{t}})S]dt. \end{array}$$

The differential equation for *S*_*t*_ can be written as

$$\frac{dS_{t}}{dt}=(\alpha -\beta K_{t})S_{t}C_{t}$$

The system of two differential equations $\frac{dK_{t}}{dt}$ and $\frac{dS_{t}}{dt}$ has an equilibrium point characterized by the following conditions

$$\begin{array}{cc} K^{*}[(\alpha +\beta )S^{*}-\gamma ] & =0\\ S^{*}[\alpha -\beta K^{*}] & =0 \end{array}$$

Thus, the equilibrium point is given by

$$K^{*}=\frac{\alpha }{\beta }\;\text{and}\;S^{*}=\frac{\gamma }{\alpha +\beta }.$$

**Fig 9:**
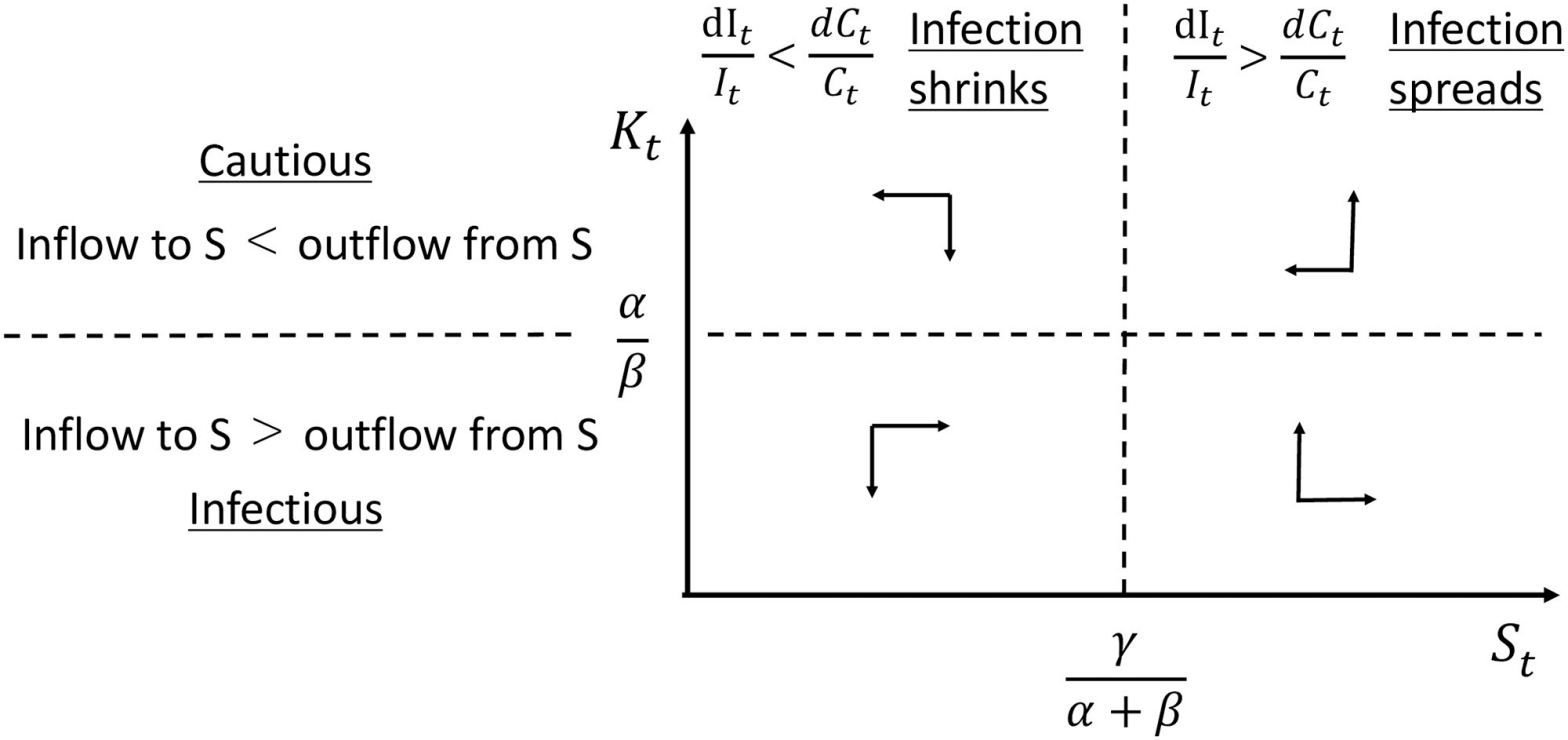
Phase diagram of *S* and $K=\frac{I}{C}$.

**Fig 10:**
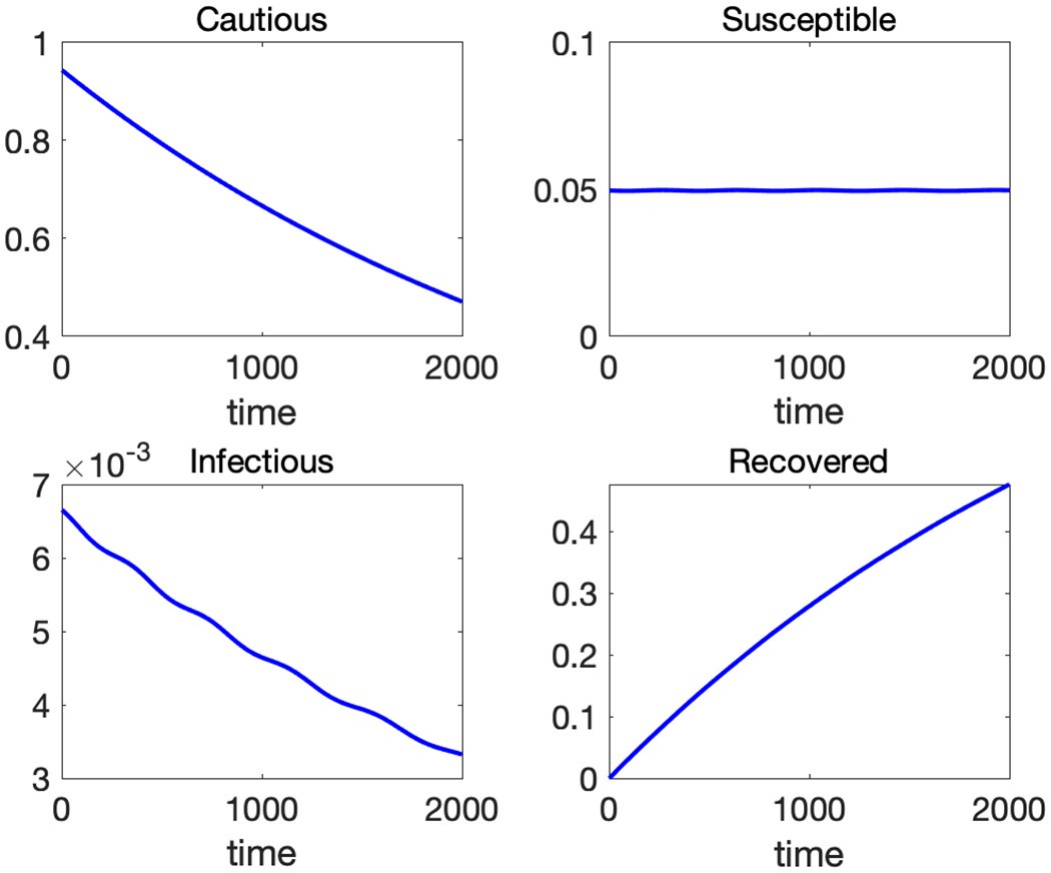
Steady-state paths of the CSIR model when $\frac{I_{0}}{C_{0}}=\frac{\alpha }{\beta }$ and $S_{0}=\frac{\gamma }{\alpha +\beta }$.

The phase diagram of this system is described in Fig 9. When *K*_*t*_ is larger than $\frac{\alpha }{\beta }$, *S*_*t*_ is decreasing, and vice versa. When *S*_*t*_ is larger than $\frac{\gamma }{\alpha +\beta }$, *K*_*t*_ is increasing, and vice versa. At the steady state $\left(S^{*},K^{*})=(\frac{\gamma }{\alpha +\beta },\frac{\alpha }{\beta }\right)$, S* remains constant and *C*_*t*_ and *I*_*t*_ are decreasing at the same rate so that $\frac{I_{t}}{C_{t}}$ is constant. Fig 10 illustrates the steady-state paths of the CSIR model when the initial conditions correspond to the equilibrium point. We see that *S*_*t*_ is constant over time, and *C*_*t*_ and *I*_*t*_ are declining at the same rate. On the steady-state paths, the differential equation of *C*_*t*_ can be solved analytically as


$$\begin{array}{c} C_{t}^{*}=C_{0}e^{-\frac{\alpha \gamma }{\alpha +\beta }t} \end{array}$$ (5)

Analogously, the path of $I_{t}^{*}$ is also given by $I_{t}^{*}=I_{0}e^{-\frac{\alpha \gamma }{\alpha +\beta }t}$.

### 3.4 CSIR model as the SIR formulation

Using the analytical results of the steady state, we can describe the CSIR model as an extended SIR model.

The dynamics of *S*_*t*_, *I*_*t*_, and *R*_*t*_ in Eqs (1)–(4) can be written as in the following SIR formulation

$$\begin{array}{c} \frac{dS_{t}}{dt}=\alpha S_{t}I_{t}^{-\frac{\alpha }{\beta }}C_{0}(I_{0}e^{-\gamma t}{)}^{\frac{\alpha }{\beta }}-\beta S_{t}I_{t} \end{array}$$ (6)

$$\begin{array}{c} \frac{dI_{t}}{dt}=\beta S_{t}I_{t}-\gamma I_{t} \end{array}$$ (7)

$$\begin{array}{c} \frac{dR_{t}}{dt}=\gamma I_{t}, \end{array}$$ (8)

where the derivation of Eq (6) is relegated to S2 Appendix.

A critical difference from the standard SIR model is the first term of Eq (6), which is complex. Because of this term, the CSIR model allows the susceptible population to increase and decrease depending on the value of *I*_*t*_. It can be verified that $\frac{dS_{t}}{dt}=0$ when *I*_*t*_ is declining at a constant rate $-\frac{\alpha \gamma }{\alpha +\beta }$ as shown in Eq (5).

If we linearize the system around the steady state $\left(S^{*},K^{*})=(\frac{\gamma }{\alpha +\beta },\frac{\alpha }{\beta }\right)$, we have

$$[\begin{array}{c} {\dot{S}}_{t}\\ \dot{K_{t}} \end{array}]=[\begin{array}{cc} 0 & -\frac{\beta \gamma }{\alpha +\beta }(\frac{\beta }{\alpha }K_{0}{)}^{\frac{\alpha }{\alpha +\beta }}C_{0}e^{-\frac{\alpha \gamma }{\alpha +\beta }t}\\ \frac{\alpha (\alpha +\beta )}{\beta } & 0 \end{array}][\begin{array}{c} S_{t}-S^{*}\\ K_{t}-K^{*} \end{array}].$$

Because diagonals of the Jacobian matrix are zero, eigenvalues are complex conjugate and this system is characterized by a periodic solution. However, because of the term $e^{-\frac{\alpha \gamma }{\alpha +\beta }t}$, as *t* → ∞, the force to generate waves will slow down and approach to zero resulting in a finite number of waves.

## 4 Comparison between SIR and CSIR models

### 4.1 Implications of a more infectious virus

This section analyzes implications of a more infectious virus in the CSIR model and contrast them to those in the SIR model.

**Fig 11:**
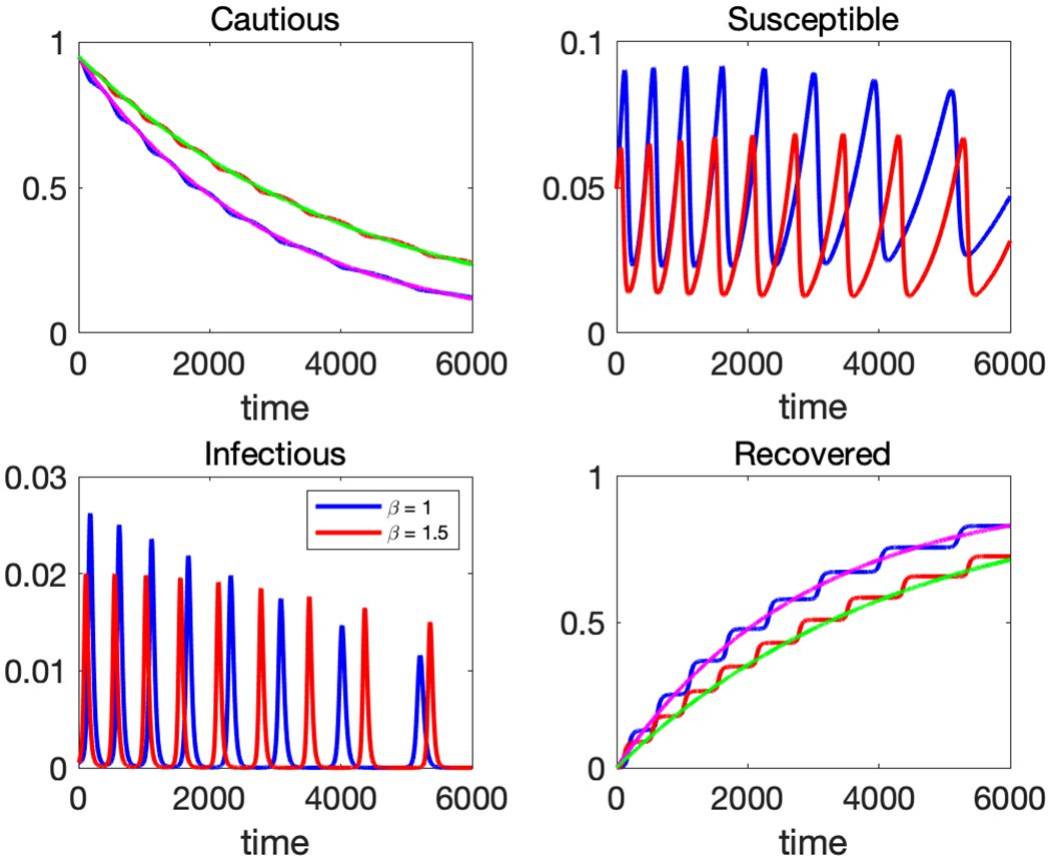
Time path of the CSIR model with *β* = 1 (blue) and *β* = 1.5 (red).

Fig 11 presents the time paths of the CSIR model with two different values of *β*. Red lines correspond to a 1.5 times higher transmission rate. Other parameter values and initial conditions are the same as in the previous section. We extend the time horizon to 6000 periods to show long-run implications.

As can be seen in the bottom-left panel, a larger *β* does not necessarily imply higher peaks of infection. In the first 2000 periods, red lines (higher *β*) exhibit lower peaks of infection. When *β* is higher, the speed of infection spread is fast and the susceptible population is exhausted sooner compared to the case of a lower *β* because the inflow from *C* cannot keep up the rapid outflow from *S*. Therefore, temporary herd immunity is achieved sooner at a lower peak. However, the height of peaks will be reversed as time passes. From the top-left panel of Fig 11, we see that the higher b scenario depletes C at a slower pace. Eventually, this will strengthen the power to pump up S, so the height of infection peaks remains elevated.

This feature—a higher beta lowers the peak values of earlier waves but increases the peak of later waves—is in contrast to what we observe in the SIR model. As we saw in Fig 2, the peak of infection is higher if *β* is larger in the standard SIR model.

Another interesting contrast between the CSIR and SIR models lies in the epidemic final size, *R*(∞) given an initial condition. In the SIR model described in Section 2, we obtain $\frac{dI}{dS}=-1+\frac{\gamma }{\beta S}$, which yields $I(t)=-S(t)+\frac{\gamma }{\beta }\;\text{ln}\;S(t)+b$ where *b* is a constant. Using *I*(∞) = 0 and assuming S(0) ≈ 1, we obtain $S(\infty )=e^{-\frac{\beta }{\gamma }[1-S(\infty )]}$, or equivalently

$$R(\infty )=1-e^{-\frac{\beta }{\gamma }R(\infty )}.$$

The final size of cumulative infected population is increasing in *β*. This can be verified in Fig 2 as well. See [3] for detailed discussions on the derivation of epidemic final sizes.

In contrast, the opposite turns out to be true in the CSIR model. On the steady-state paths of the CSIR model,

$$\begin{array}{c} \frac{dR^{*}(t)}{d\beta }<0, \end{array}$$ (9)

so more infectious virus leads to a smaller size of cumulative infected population at any given time (see S3 Appendix for proof).

Holding *α* and *γ*, an increase in *β* leads to smaller cumulative infection as can be seen in the bottom-right panel of Fig 11. As discussed above, a highly infectious virus (a higher *β*) exhausts the susceptible population too quickly compared to the rate of inflow from the cautious group, and hence, does not result in more cumulative infection. In the bottom-left panel of the same figure, the area below the infection curves determines to cumulative infection. The infection wave of a higher *β* exhibits steeper shapes and a shorter period (more waves within a fixed time window), so the area under the red curve is smaller than that under the blue curve.

In Fig 11, we overlay the steady-state paths of *C* and *R* as purple and green lines. They are computed from the equations shown in subsection 3.3 with the same parameter set, but initial values are replaced with the steady-state values. Given a set of parameters, the paths of *C* and *R* can be approximated by the steady-state paths, and the waves of *I* can be considered as a perturbation from those paths. If the initial conditions are close to the steady state values $\left(S^{*},K^{*})=(\frac{\gamma }{\alpha +\beta },\frac{\alpha }{\beta }\right)$, the system generates many waves. This explains the contour plot of the number of waves in Fig 7. With the initial values *C*(0) = 0.95, *S*(0) = 0.0495, *I*(0) = 0.0005, and *β* = 1, when $\alpha =\frac{0.0005}{0.95}=0.000526$ and *γ* = *S*(0) × (*α* + *β*) = 0.0495 × 1.000526 = 0.049526, the initial conditions perfectly coincide the steady state values. Thus, around those parameter values of *α* and *γ*, we observe many waves.

### 4.2 A large wave versus small multiple waves

**Fig 12:**
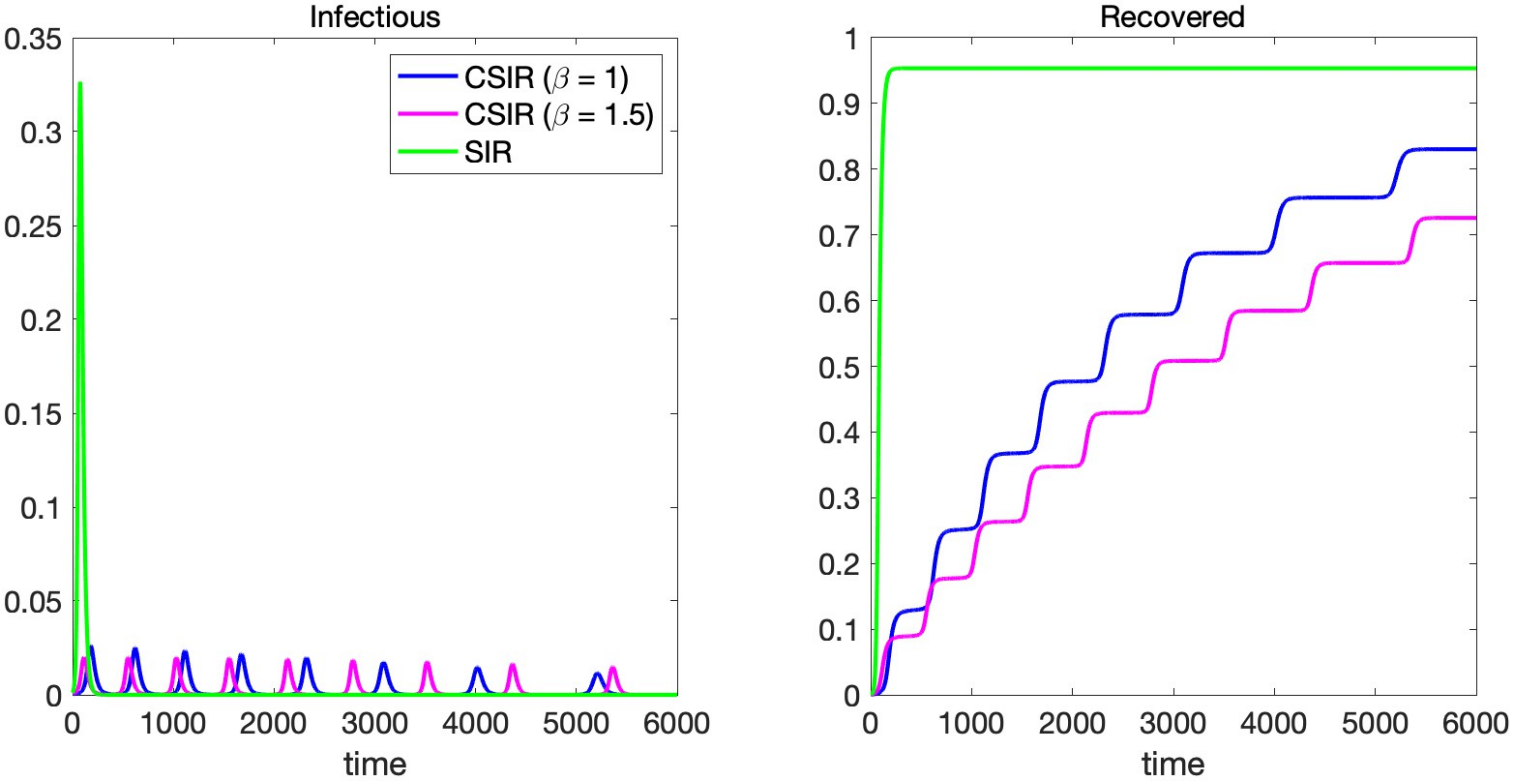
The paths of the infectious and recovered in CSIR and SIR models.

Another contrast between SIR and CSIR models is the dynamics of infection over time. In Fig 12, the paths of the infectious and recovered are depicted for SIR and two cases of CSIR models (*β* = 1 and *β* = 1.5). Other parameters are set so that the final size will be equal for all models. The final size of the CSIR model is determined by *C*_0_ + *I*_0_ as described in S3 Appendix. In the figure, we show the time paths in the first 6000 periods. The SIR model generates a large wave of infection in the early phase of pandemic, and quickly reaches to the herd-immunity steady state. In this parameter specification, the number of new cases reaches more than 30% of total population at its peak, and about 95% of total population get infected in the very early phase. On the other hand, the CSIR model generates many small waves of infection over time, which is closer to reality as shown in Fig 1.

## 5 Other theories of waves

In this section, we review other theories of waves in SIR-like compartment models to be precise about our contribution. [4] reviews some of the papers in this section in detail. Beyond the SIR model, some have shown that network models of infection—such as scale-free and small-world networks—can generate multiple waves. See, for example, [5].

Our paper is closely related to [6], who consider the contagion dynamics of both fear and disease. In the model, pathogen (disease) spreads in a similar manner to the SIR model, but fear for disease also spreads among the population. Individuals can “contract” fear through contact with individuals who are infected with the disease (the sick) or infected with fear (the scared). Some of the scared individuals remove themselves from circulation and do not get sick. This self-isolation grants rich epidemic dynamics to the model, which can explain multiple waves of infection. [7] extend this model by incorporating the fear for vaccine. [8] propose a similar compartment model to ours which incorporates agents’ voluntary quarantine strategies. Their model can also generate infection waves, but it has five compartments with richer interactions based on perceived payoffs in the game theory. [9, 10] review other types of game-theoretic epidemiological frameworks. Although our CSIR model has a similar mechanism to these models, it is much simpler, yet contains sufficient elements to explain waves of infection. We believe that our parsimonious framework with an endogenous behavioral change can serve as a basis for the analysis of realistic infection paths.

Other theories also focus on people’s behavioral change, but only some of them can explain infection waves. The basic mechanism is the same across those theories. As the number of infections and deaths increases, people restrain their activity or take appropriate prevention measures so as to reduce the risk of infection. Accordingly, when the number of infections or deaths increases sufficiently, infection starts declining. As infection or death counts fall, people start relaxing preventive measures, which eventually leads to a rise in infection. Models in which the transmission rate inversely depends on infection or deaths are consistent with this story, but it is not enough to generate infection waves. [11, 12] show that it is not possible to generate waves in SIR models in which the transmission rate continuously depends on *I*(*t*) in certain functional forms. [13] show that it is possible to generate waves if the transmission rate is a jump function of *I*(*t*). [14] show that it is possible to generate waves in a model where the current force of infection depends on the past level of infected population. [15] incorporate behavioral response in population to an SEIRS model to analyze the COVID-19 pandemic. Their model may also generate infection waves.

Some theories emphasize the role of government policies in promoting people’s behavioral changes. When medical resources are limited, a government may need to impose NPIs to avoid collapse of medical system when infection rises and lift NPIs when infection declines and medical pressures loosen. This cycle can also create waves as investigated in [16, 17]. Empirical literature distinguishes voluntary and forced lockdown/social-distancing, with some arguing that the former was more important than the latter during the COVID-19 pandemic. See, for example, [18, 19].

Models with the possibility of reinfection can also generate waves. One class of models with reinfection is the SIRS model in which recovered people probabilistically lose their immunity and become susceptible again. The existing studies have shown that SIRS models can generate waves under certain conditions, though they do not necessarily generate waves. See [20–22] for the applications of SIRS models to COVID-19. [23] incorporate government action and public response to a SIRS model. Another class of models with reinfection is age-structured model in which the effect of vaccines wane over time. [24–26], among others, study conditions under which periodic solutions emerge.

A more straightforward way to generate waves is to exogenize the transmission rate and make it seasonal ([14, 27]). In a similar vein, periodic appearance of a more infectious variant—which can be captured by making the transmission rate increases over time in a step function—is likely to be able to generate waves.

Our model differs from these existing theories of waves because it features heterogeneity in risk attitudes and their time-variation. Waves during the COVID-19 pandemic are likely to reflect a mixture of these various forces. If new agents are continuously born, it is possible for the SIR model to generate periodic solutions, but this does not account for what we have observed during the COVID-19 pandemic because multiple waves occurred within a year or two. Which forces were more dominant in reality likely depends on time and place. It would be useful to quantitatively investigate such a question in future research.

## 6 Conclusion

Many countries have experienced multiple waves of infection during the COVID-19 pandemic. In this paper, we have presented a novel extension of the SIR model, the CSIR model, that can endogenously generate such multiple waves. Key features of our model are heterogeneity in risk attitudes among those who have not been infected and time-variation in these risk attitudes. We think that the mechanism in our model is a plausible contributing factor to the emergence of multiple waves during the COVID-19 pandemic and that it complements other theories of waves.

It would be interesting to examine the role of factors that are likely to have been important during the COVDI-19 pandemic, such as the role of vaccines and NPIs, in our CSIR model framework. It would be also interesting to extend the model to analyze the joint dynamics of infection and economy—as done in the economics literature using macro-SIR models—and examine the effects of various policy interventions on both infection and economy. See, for example, [16, 28–33], among many others. We leave these interesting extensions for future research.

## Supporting information

S1 Appendix Alernative specifications. (PDF)

S2 Appendix Derivation of equation (6). (PDF)

S3 Appendix Proof of equation (9). (PDF)

S1 File (ZIP)
