# Supplementary material for: Heterogeneous risk attitudes and waves of infection
Source: PLoS One. 2024 Apr 9;19(4):e0299813. doi: 10.1371/journal.pone.0299813 (PMC11003633; doi:10.1371/journal.pone.0299813)
Supplement: S1 Appendix — (PDF) [file pone.0299813.s001.pdf]

S1Appendix
Alternative Specifications

The flow between the cautious and susceptible determines the dynamics of the CSIR model. In the main text, we present a particular specification, but infection waves can occur in other specifications. The laws of motion for C and S can be described by the following equations

$$\frac{dC(t)}{dt} = -FlowCS(t)$$
$$\frac{dS(t)}{dt} = FlowCS(t) - \beta S(t) I(t).$$

Consider the following cases where $FlowCS(t)$ are characterized by

$$\text{Model 1}: \alpha C(t) S(t)$$
$$\text{Model 2}: \alpha C(t) S(t) - \theta S(t) I(t)$$
$$\text{Model 3}: \alpha C(t) S(t) - \theta I(t)$$
$$\text{Model 4}: \alpha C(t)$$
$$\text{Model 5}: \alpha C(t) - \theta S(t)$$
$$\text{Model 6}: \alpha C(t) - \theta C(t) S(t)$$
$$\text{Model 7}: \alpha C(t) - \theta S(t) I(t).$$

Model 1 corresponds to our baseline specification presented in Section 3. Models 2 and 3 allow a reverse flow from S to C, which is increasing in the current infectious populations. These models intend to capture the "fear" effect, by which a certain fraction of the susceptible become cautious again when infection spreads in the society. Models 4 through 7 assume that a constant fraction of the cautious individuals become susceptible over time regardless of the current susceptible population (no peer effect). Models 5 through 7 allow a reverse flow from S to C in different forms.

Figures 1 and 2 show that infection waves can be generated in all models. In Model 2, a temporary increase of the cautious can be observed, which implies a monotonic decline of C is not a necessary condition to generate the waves of infection. Of course, the generation of waves are not guaranteed for any parameter values, but we can find a set of parameters to generate waves in all specifications.

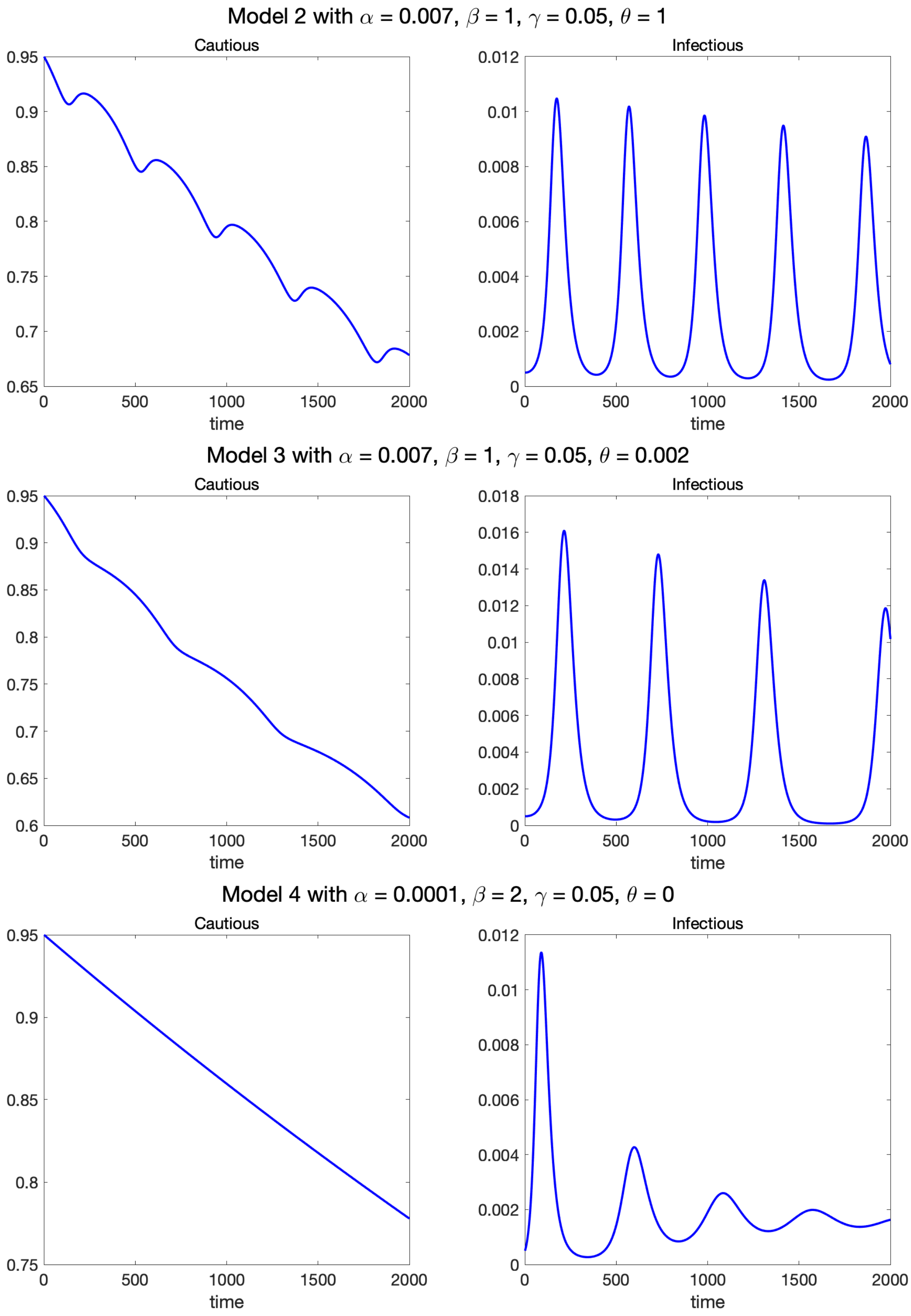

Fig 1. Infection waves in Models 2, 3, 4

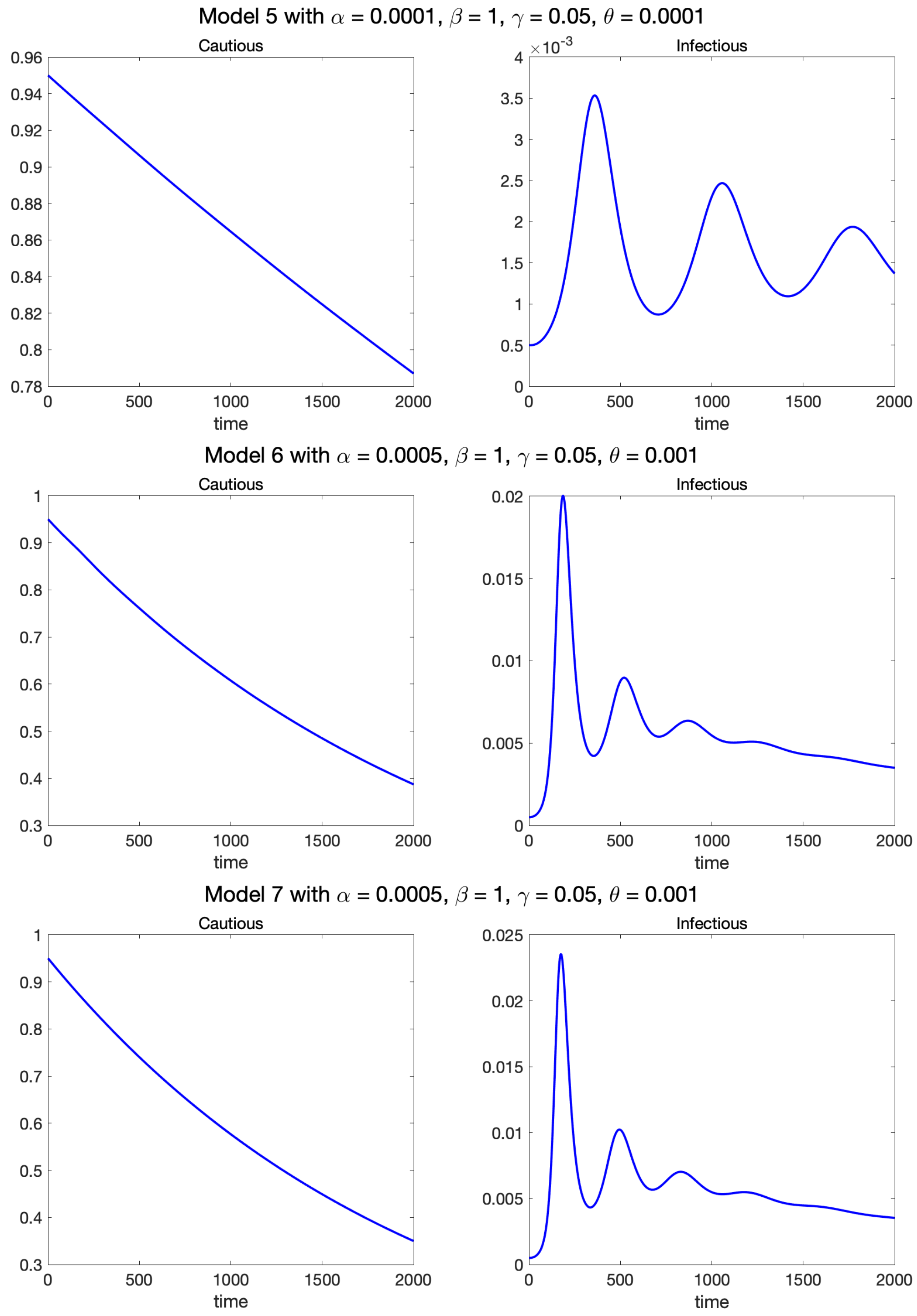

Fig 2. Infection waves in Models 5, 6, 7
