# Supplementary material for: Heterogeneous risk attitudes and waves of infection
Source: PLoS One. 2024 Apr 9;19(4):e0299813. doi: 10.1371/journal.pone.0299813 (PMC11003633; doi:10.1371/journal.pone.0299813)
Supplement: S2 Appendix — (PDF) [file pone.0299813.s002.pdf]

S2 Appendix
Derivation of Equation (6)

At the steady state paths,

$$C_t = C_t^*, S^* = \frac{\gamma}{\alpha+\beta}, K^* = \frac{\alpha}{\beta}.$$

Define a deviation of C_t from the steady state path as $\varepsilon_t = \frac{C_t}{C_t^*}$. Then,

$$d\varepsilon_t = d\left(\frac{C_t}{C_t^*}\right) = \frac{dC_t C_t^* - C_t dC_t^*}{C_t^{*2}} = \frac{\left(-\alpha S_t C_t\right) C_t^* - C_t\left(-\frac{\alpha\gamma}{\alpha+\beta}C_t^*\right)}{C_t^{*2}}dt = \left(-\alpha S_t \frac{C_t}{C_t^*} + \frac{\alpha\gamma}{\alpha+\beta}\frac{C_t}{C_t^*}\right)dt,$$

which can be written as

$$\frac{d\varepsilon_t}{dt} = \alpha\left(\frac{\gamma}{\alpha+\beta} - S_t\right)\varepsilon_t.$$

Hence, we obtain the following system

$$\begin{aligned}
\frac{d\varepsilon_t}{dt} &= \alpha\left(\frac{\gamma}{\alpha+\beta} - S_t\right)\varepsilon_t \\
\frac{dS_t}{dt} &= \left(\alpha - \beta K_t\right) C_0 \varepsilon_t S_t e^{-\frac{\alpha\gamma}{\alpha+\beta}t} \\
\frac{dK_t}{dt} &= \left[\left(\alpha+\beta\right) S_t - \gamma\right] K_t
\end{aligned}$$

We have $\frac{dK_t}{\gamma K_t dt} = \frac{(\alpha+\beta)}{\gamma}S_t - 1$ and $\frac{d\varepsilon_t}{\alpha\varepsilon_t dt} = \frac{\gamma}{\alpha+\beta} - S_t$. Then,

$$\begin{aligned}
&\frac{\alpha+\beta}{\alpha\gamma}\frac{d\varepsilon_t}{\varepsilon_t dt} = 1 - \frac{(\alpha+\beta)}{\gamma}S_t = -\frac{dK_t}{\gamma K_t dt} \\
&\Rightarrow \frac{\alpha+\beta}{\alpha\gamma}\ln\varepsilon_t = -\frac{1}{\gamma}\ln K_0 + C \Rightarrow 0 = \frac{\alpha+\beta}{\alpha\gamma}\ln\varepsilon_0 = -\frac{1}{\gamma}\ln K_0 + C \\
&\Rightarrow C = \frac{1}{\gamma}\ln K_0 = \frac{1}{\gamma}\ln\frac{I_0}{V_0} \Rightarrow \frac{\alpha+\beta}{\alpha\gamma}\ln\varepsilon_t = -\frac{1}{\gamma}\ln K_t + \frac{1}{\gamma}\ln K_0 \\
&\Rightarrow \frac{\alpha+\beta}{\alpha}\ln\varepsilon_t = \ln\frac{K_0}{K_t} \Rightarrow \varepsilon_t^{\frac{\alpha+\beta}{\alpha}} = \frac{K_0}{K_t}
\end{aligned}$$

Thus,

$$\varepsilon_t = \left(\frac{K_0}{K_t}\right)^{\frac{\alpha}{\alpha+\beta}}$$

Because $\varepsilon_t = \frac{C_t}{C_t^*}$,

$$\begin{aligned}
C_t &= C_t^{*\frac{\alpha+\beta}{\beta}}\left(\frac{I_0}{C_0}\right)^{\frac{\alpha}{\beta}} I_t^{-\frac{\alpha}{\beta}} = \left(C_0 e^{-\frac{\alpha\gamma}{\alpha+\beta}t}\right)^{\frac{\alpha+\beta}{\beta}}\left(\frac{I_0}{C_0}\right)^{\frac{\alpha}{\beta}} I_t^{-\frac{\alpha}{\beta}} \\
&= C_0\left(\frac{I_0}{I_t}e^{-\gamma t}\right)^{\frac{\alpha}{\beta}}
\end{aligned}$$

The differential equation of S_t can be written as

$$\begin{aligned}
\frac{dS_t}{dt} &= \alpha S_t C_t - \beta I_t S_t = \alpha S_t C_0\left(\frac{I_0}{I_t}e^{-\gamma t}\right)^{\frac{\alpha}{\beta}} - \beta I_t S_t \\
&= \alpha S_t I_t^{-\frac{\alpha}{\beta}} C_0\left(I_0 e^{-\gamma t}\right)^{\frac{\alpha}{\beta}} - \beta S_t I_t
\end{aligned}$$

which concludes the derivation.
