# Supplementary material for: Heterogeneous risk attitudes and waves of infection
Source: PLoS One. 2024 Apr 9;19(4):e0299813. doi: 10.1371/journal.pone.0299813 (PMC11003633; doi:10.1371/journal.pone.0299813)
Supplement: S3 Appendix — (PDF) [file pone.0299813.s003.pdf]

S3 Appendix
Proof of Equation (9)

On the steady-state path,C_t^* and I_t^* evolve as

$$C_t^* = C_0 e^{-\frac{\alpha\gamma}{\alpha+\beta}t}$$
$$I_t^* = \frac{\alpha}{\beta} C_t^* = \frac{\alpha}{\beta} C_0 e^{-\frac{\alpha\gamma}{\alpha+\beta}t}$$

Because the differential equation of R_t is given by

$$\frac{dR_t}{dt} = \gamma I_t,$$

at the steady state, it can be written as

$$\frac{dR_t^*}{dt} = \gamma \frac{\alpha}{\beta} C_0 e^{-\frac{\alpha\gamma}{\alpha+\beta}t}$$

Integrating both sides yields

$$R_t^* = -\gamma I_0 \frac{\alpha+\beta}{\alpha\gamma}\left(e^{-\frac{\alpha\beta}{\alpha+\beta}t} - 1\right) = I_0 \frac{\alpha+\beta}{\alpha}\left(1 - e^{-\frac{\alpha\gamma}{\alpha+\beta}t}\right)$$

Since $I_0 = \frac{\alpha}{\beta} C_0$, we can also write the above equation as

$$R_t^* = (C_0 + I_0)\left(1 - e^{-\frac{\alpha\gamma}{\alpha+\beta}t}\right)$$

Differentiating R_t^* with respect to β yields

$$\frac{dR_t^*}{d\beta} = -\frac{\alpha\gamma}{(\alpha+\beta)} t \left(C_0 + I_0\right) e^{-\frac{\alpha\gamma}{\alpha+\beta}t} < 0,$$

which concludes the derivation.
